# Possible Roles of Interleukin-4 and -13 and Their Receptors in Gastric and Colon Cancer

**DOI:** 10.3390/ijms22020727

**Published:** 2021-01-13

**Authors:** Xujun Song, Benno Traub, Jingwei Shi, Marko Kornmann

**Affiliations:** Department of General and Visceral Surgery, Ulm University Hospital, Albert-Einstein-Allee 23, 89081 Ulm, Germany; 101012268@seu.edu.cn (X.S.); benno.traub@uniklinik-ulm.de (B.T.); shijingwei555@126.com (J.S.)

**Keywords:** interleukin-4, interleukin-13, interleukin-4 receptor, interleukin-13 receptor, gastrointestinal cancer

## Abstract

Interleukin (IL)-4 and -13 are structurally and functionally related cytokines sharing common receptor subunits. They regulate immune responses and, moreover, are involved in the pathogenesis of a variety of human neoplasms. Three different receptors have been described for IL-4, but only IL-4 receptor type II (IL-4Rα/IL-13Rα1) is expressed in solid tumors. While IL-13 can also bind to three different receptors, IL-13 receptor type I (IL-4Rα/IL-13Rα1/IL-13Rα2) and type II (IL-4Rα/IL-13Rα1) are expressed in solid tumors. After receptor binding, IL-4 and IL-13 can mediate tumor cell proliferation, survival, and metastasis in gastric or colon cancer. This review summarizes the results about the role of IL-4/IL-13 and their receptors in gastric and colon cancer.

## 1. Introduction

Gastric cancer (GC) and colon and rectal cancer (CRC) are common malignancies of the digestive system [1,2]. Despite advances in earlier detection, multimodal treatment, and surgical management, the prognosis of both entities is still unsatisfactory [3]. CRC is the second leading cause of all tumor deaths in the United States [3], and stomach cancer is the third leading death cause of cancer-related deaths worldwide [4]. Alternative or additional treatment strategies especially for advanced tumor stages are desperately needed to overcome drug resistance, enhance chemosensitivity, inhibit tumor cell proliferation, and induce apoptosis in order to further improve outcome [1,2,5,6,7,8,9].

More and more evidence has been provided in recent years that interleukin-4 (IL-4), interleukin-13 (IL-13), and their receptors play an important role in cancer cell proliferation and other biological behaviors, such as migration and invasion enhancing the malignant phenotype [10,11,12]. Moreover, IL-4/IL-13 and their receptors have been also associated with apoptosis, chemosensitivity, and prognosis in various cancers [13,14,15]. IL-4 and IL-13 are also involved in the crosstalk with the tumor microenvironment (TME) by activating tumor-associated macrophages and myeloid-derived suppressor cells, which have tumor promoting functions [16,17]. Immune surveillance against established metastatic mammary cancer is negatively regulated by IL-13 in mice [18]. The aim of this review was to summarize the current information about the role of IL-4/IL-13 and their receptors in GC and CRC.

## 2. Methods

A literature search in PubMed was carried out in April 2020 using “interleukin-4” or “interleukin-13” or “interleukin-4 receptor” or “interleukin-13 receptor” in combination with “gastric cancer” or “colon cancer” or “colorectal cancer” or “rectal cancer”, respectively. A total number of 452 articles were retrieved. Duplicate articles were eliminated, and additional articles were identified through references cited in retrieved articles. Only manuscripts and reviews in the English language were included in this review.

The articles about single nucleotide polymorphisms (SNPs) were searched in May 2020 using “interleukin-4” or “interleukin-13” or “interleukin-4 receptor” or “interleukin-13 receptor” and “polymorphism OR mutation OR variation” in combination with “colorectal cancer” or “colon cancer” or “rectal cancer”. Sixty-four articles about SNPs of IL-4/IL-13 and their receptors in CRC were identified. A total of 67 articles about SNPs of IL-4/IL-13 and their receptors in GC were retrieved using “interleukin-4” or “interleukin-13” or “interleukin-4 receptor” or “interleukin-13 receptor” and “polymorphism OR mutation OR variation” and “gastric cancer”. Our review included a total of 64 articles about SNPs of IL-4/IL-13 and their receptors in GC or CRC.

## 3. Summary of the IL-4/-13 Signaling Pathway

IL-4, first described in 1981, is a secreted cytokine that, in its physiologic function, can regulate antibody production, hematopoiesis, and inflammation, and is also involved in the development of effector T-cell responses [19]. The closely related IL-13, first described in 1993, is a human lymphokine that can regulate inflammatory and immune responses [20]. IL-4 and IL-13 are essential for the induction and persistence of the type 2 immune response, and they are associated with multiple atopic diseases, such as asthma and atopic dermatitis [21]. IL-4 and IL-13 are mainly produced by immune cells, such as CD4-T-cells, basophils, eosinophils, and natural killer T (NKT) cells [22].

**Figure 1:**
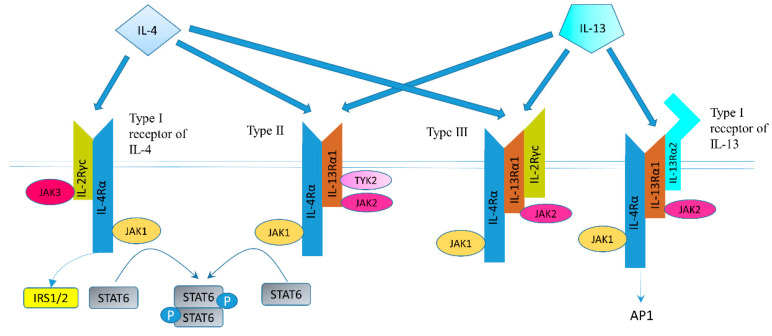
Receptor types and signal transduction of IL-4R and IL-13R. There are three different kinds of IL-4 receptors. IL-4 binds to the IL-4Rα chain, then recruits the IL-2Rγ-common (γc) chain (type I IL-4R) or the IL-13Rα1 chain (type II IL-4R) to form a receptor complex that can initiate signal transduction. The type III IL-4R consists of all three chains. IL-13 also have three different receptors. IL-13 receptor type I (IL-4Rα/IL-13Rα1/IL-13Rα2) and type II (IL-4Rα/IL-13Rα1) are expressed in solid tumors, while type III (IL-4Rα/IL-13Rα1/γc) is expressed in hemocytes only. IL-13 binds IL-13Rα1 with a low affinity and complexes with the IL-4Rα (type II receptor). IL-13 binds to the IL-13Rα2 with a high affinity. IL-13 can bind to a soluble IL-13Rα2 receptor, which has no downstream signaling, or bind to transmembrane IL-13Rα2 and activate AP-1. Figure sketch adapted from reference [27].

The structure of IL-4 receptor (IL-4R), IL-13 receptor (IL-13R), and the positions of the intracellular signaling molecules of them have been summarized in several articles [23,24,25,26]. There are three different kinds of IL-4 receptors (Figure 1). IL-4 binds to the IL-4Rα chain, then recruits the IL-2Rγ-common (γc) chain (type I IL-4R) or the IL-13Rα1 chain (type II IL-4R) to form a receptor complex that can initiate signal transduction [25]. The type III IL-4R is formed by all the three chains [27]. IL-13 can also signal via three different receptors (Figure 1). The type II IL-13R complex has the same components as the type II IL-4R [28]. IL-13R type I (IL-4Rα/IL-13Rα1/IL-13Rα2) and type II (IL-4Rα/IL-13Rα1) are expressed in non-hematopoietic cells, while type III (IL-4Rα/IL-13Rα1/γc) is only expressed on the surface of hemocytes [27]. Overall, this results in a possible complex web of IL-4– and IL-13–mediated signaling pathways [29].

With regard to the IL-13Rα2 chain, IL-13 is not the sole ligand. For example, chitinase-3-like protein 1 (CHI3L1) could bind to IL-13Rα2 and regulate oxidant injury, apoptosis, and melanoma metastasis [30]. Transmembrane protein 219 and CD44 play an important role in IL-13Rα2 mediated signaling which is induced by CHI3L1 [31,32].

Altogether, IL-4R and IL-13R share two receptor chains (IL-4Rα and IL-13Rα1) and can mediate common, but also diverse biological functions [27]. Both IL-4 and IL-13 phosphorylate and activate signal transducer and activator of transcription (STAT) 6 [27]. STAT3, STAT5, and STAT1 can also be activated, but to a lesser degree [23]. IL-4 can signal through IRS-2 (generally expressed by hematopoietic cells) or IRS-1 (generally non-hematopoietically expressed) [23]. As mentioned above, IL-13 could bind to the IL-13Rα2 chain, which has a very high affinity for IL-13. The downstream signaling involves AP-1 family members c-jun and Fra-2 [33]. IL-13Rα2 can inhibit downstream signals of IL-13R and IL-4R through regulating STAT6 [34,35].

## 4. IL-13/IL-13R in Gastric Cancer

IL-13Rs are overexpressed in several human solid cancer cell lines [36,37]. Our group demonstrated that IL-13R and IL-4R were expressed in pancreatic cancer cell lines, such as PANC-1, MIAPaCa-2, and CAPAN-1 [38]. Their proliferation was inhibited by Pseudomonas exotoxin (PE) combined to IL-13 or IL-4, demonstrating the receptor’s functionality [38]. IL-13Rα2 is expressed in HS766T and MIAPaCa-2 pancreatic cancer cells, as well [36]. One recombinant chimeric protein IL-13PE was found highly cytotoxic to GC cell line CRL1739, which also expressed the type II IL-4R receptor (Figure 1) binding both IL-4 and IL-13 [39]. IL-13Rα2 is also expressed in GC cell lines MKN-45, AGS and MGC308 [32,40].

Gabitass et al. evaluated plasma IL-13 and IL-4 levels in 131 patients (46 pancreatic cancer, 25 GC, and 60 esophageal cancer) and 54 healthy controls [41]. IL-13 levels in patients’ plasma were significantly higher in all the three cancer patients compared with controls [41]. In another study, Lin et al. evaluated IL-13Rα2 expression in tissue microarrays of 507 GC patients [15]. They found the overexpression of the IL-13Rα2 chain in cancer tissue was associated with poor prognosis after gastrectomy [15].

Chen and coworkers showed that CHI3L1 secreted by M2 macrophage could promote the metastasis of GC cell lines MKN-45 and AGS by binding to the IL-13Rα2 chain [40]. The mechanism is mediated by activating the mitogen-activated protein kinase signaling pathway, which upregulates the matrix metalloproteinase genes [40]. Geng et al. found CD44v3 could bind to both CHI3L1 and IL-13Rα2 in GC cell lines AGS and MGC308 [32]. In this study, CHI3L1 expression was positively related to GC invasion depth and lymph node status in 100 GC tissues from patients [32]. A summary of the results is shown in Supplemental Table S1.

## 5. IL-4/IL-4R in Gastric Cancer

Human GC cell lines such as CRL1739 express IL-4R [42]. IL-4 inhibited proliferation of HTB-135 GC cells by down-regulating G0-G1 cell cycle nuclear-regulating factors, including retinoblastoma gene product, c-myc, and cyclin D1 [43]. IL-4 could cause G1 phase arrest in the GC cell line CRL 1739 by binding to IL-4Rα and γc (type I IL-4R) [42]. IL-4 could also inhibit the growth of GC cells and this effect was positively related with IL-4R expression level of the respective cell lines [44]. The expression was detected by flow cytometry using biotin-labeled IL-4. It remains unclear, however, what type of IL-4R causing inhibition of GC cell growth was expressed (Figure 1).

Gabitass et al. found plasma IL-4 levels in 25 GC patients were significantly higher than in 54 healthy controls [41]. Cárdenas et al. found serum IL-4 levels in 17 GC patients were significantly elevated comparing with 30 healthy individuals by sandwich ELISA [45]. In their study, elevated serum levels of IL-4 indicated a higher risk of mortality, but there is no statistical association [45]. Orea and co-workers studied a total of 30 biopsies of GC by immunohistochemistry [46]. They found a significantly higher expression of IL-4 in stages I and II than in stages III and IV, pointing to a possible growth inhibitory effect of IL-4 in GC [46]. A summary of the effects of IL-4 and IL-13 in GC is shown in Supplemental Table S1.

## 6. IL-13/IL-13R in Colon and Rectal Cancer

*Expressions of IL-13/IL-13R in CRC:* Immunoblot analysis demonstrated a high expression of IL-13Rα2 in cultured metastatic colon cancer (CC) cell lines such as KM12SM, SW48, and HT29 [13]. Overexpression of IL-13Rα2 was found in 66% of tumor samples from 80 CC patients [13]. In an immunohistochemical analysis of CRC patients at stage I–III, high IL-13 and IL-13R expression was seen in 50% (181/359) and 42% (152/359) of the cancers, respectively [47].

*IL-13/IL-13R in CRC cell lines:* IL-13 and IL-4 stimulated mucin 2 expression in CC cell line LS174T, but not in CC cell line HT-29 through the mitogen-activated protein kinase pathway [48]. IL-13 could inhibit the autophagic pathway in HT-29 cells via the activation of the class I phosphatidylinositol 3-kinase (PI3K) [49]. The tumor suppressor phosphatase and tensin homolog (PTEN) is expressed in HT-29 cells. Its overexpression directed by an inducible promoter counteracted the IL-13 down-regulation of macroautophagy, which is the most prevalent form of autophagy [50]. One recombinant chimeric protein IL-13PE was cytotoxic to CC cell lines Colo201 and Colo205 [39]. Another study revealed that IL-13 induced phosphorylation of Janus kinase (JAK) 2, JAK1, and Tyk2 in CC cell lines HT-29 and WiDr [51]. In addition, both IL-13 and IL-4 could induce phosphorylation of STAT6 [51]. In human colonic epithelial cell lines, IL-13 and IL-4 upregulated the expression of CD44 [52]. Bartolomé et al. demonstrated that family with sequence similarity 120A (FAM120A) in the IL-13/IL-13Rα2 signaling pathway was an important mediator of invasion and liver metastasis using CC cell lines such as SW620, KM12C, and KM12SM, and nude mice that were inoculated with CC cells in the spleen [11]. KM12C and KM12SM human CC cells only differ in their metastatic properties [53]. Bartolomé et al. found that FAM120A could mediate the IL-13Rα2-induced activation of the FAK and PI3K/AKT/mTOR pathways [11]. FAM120A could function as a scaffold protein for PI3K to be phosphorylated by Src family kinases [11]. Propofol suppressed cell proliferation and IL-13 induced epithelial–mesenchymal transition (EMT) in CRC cell lines RKO and SW480 [54]. It could be demonstrated that propofol suppressed IL-13/STAT6 signaling by upregulating STAT6 targeting miRNAs [54]. IL-13 promoted EMT and aggressiveness of HT-29 and SW480 cells through IL-13Rα1/STAT6/ZEB1 pathway [55]. IL-13 was found to induce the expression of 11β-hydroxysteroid dehydrogenase type 2 (11βHSD2) via IL-13Rα2 in CRC cell line SW480 and murine CRC cell line CT26 [56].

*IL-13/IL-13R in CRC mouse models:* Glycyrrhizic acid, an inhibitor of 11βHSD2, could reduce liver metastasis of CT26 cells in nude mice [56]. Protein tyrosine phosphatase-1B mediates IL-13-induced cancer cell proliferation, migration, and survival via Src activation [57]. IL-4 and IL-13 both up-regulated the expression of chemokine eotaxin-2 in CRC cell lines LS174T and LOVO [58]. However, this effect was not seen in CRC cell lines SW480 and COLO 205 [58]. More than a 10-fold increase of eotaxin-2 level was found in tissue-derived supernatants from colorectal hepatic metastases compared with normal liver in 23/25 patients [58].

Matsui et al. studied the roles of inflammatory cytokines in obesity-related colorectal tumorigenesis [59]. Colorectal tumorigenesis was induced through intraperitoneal injection of azoxymethane in C57BL mice and obesity diabetes model mice KK and KK-Ay [59]. The group found that the formation of CRC was more frequent in obese mice than wild type mice [59]. Moreover, silencing IL-13Rα1 with small interfering RNA inhibited IL-13-induced proliferation in the CC cell line HT29 via downregulating STAT6 activation [59]. Another study found that the development of lung metastases could be significantly inhibited by an IL-13 inhibitor, but not by inhibition of IL-4 in a murine lung metastasis model of CC [60]. High expression of IL-13Rα2 in the CC cell line KM12 was associated with invasion and liver metastasis in nude mice [13]. The mechanism might be the activation of the oncogenic signaling molecules such as PI3K and AKT by IL-13 [13]. High expression of IL-13Rα2 was also associated with higher tumor stages and poor outcome in human CRC patients [13]. The IL-13Rα2 D1 peptide inhibited proliferation, migration, and invasion in KM12SM and SW620 CC cells treated with IL-13 [61]. This peptide could block the signaling through IL-13Rα2 and, at a lower level, IL-13Rα1 [61]. Nude mice treated with the enantiomer D-D1 peptide had a significant longer survival time due to reduced development of liver metastasis [61].

*IL-13/IL-13R in CRC patients:* Ingram et al. studied the role of type II IL-4R in transgenic mouse models and human cases. They found reduced IL-4R increased CRC initiation but reduced tumor progression and did not show any effects on mortality [62]. In an immunohistochemical study including 359 CRC samples, patients who had high IL-13R expression had less lymph node metastases [47]. High IL-13 expression was associated with a longer survival time [47]. Saigusa et al. studied 241 patients with CRC and demonstrated that serum IL-13 levels were significantly lower in patients with advanced stage, and low IL-13 levels in the serum was significantly associated with poorer prognosis [63]. However, in a study measuring IL-13 protein levels in fecal samples, 20 CRC patients presented significantly higher IL-13 levels than 20 healthy controls [64].

A summary of the effects of IL-13/IL-13R in CRC cells are depicted in Supplemental Table S2 and the effects of IL-13/IL-13R in CRC mouse models or patients are depicted in Supplemental Table S3.

## 7. IL-4/IL-4R in Colon Cancer

*Expressions of IL-4/IL-4R in colon cancer:* Lahm et al. studied the expression of IL-4R in 7 CRC cell lines [65]. Fluorescent-activated cell sorting analysis showed that three cell lines (WiDr, LS1034, HT29,) had a relatively higher expression of IL-4R, while the other four cell lines (Co-115, LS513, SW1116, LS4llN) had lower expression [65]. Higher expression levels of IL-4 and IL-13 were found in the serum or the tumor homogenates of a CT26 tumor-bearing mouse model [66]. In an analysis of IL-4R expression in 44 CRC patients using immunohistochemistry, positive labelling was obtained in 40/44 carcinomas [67]. In an immunohistochemical study of CRC patients at stage I–III, high IL-4 and IL-4R expression were detected in 33% (118/359) and 36% (129/359) of the samples, respectively [47].

*IL-4/IL-4R in colon cancer cell lines* Liu and colleagues found IL-4 and IL-13 increased nicotinamide adenine dinucleotide phosphate oxidase 1-related proliferation in HT-29 and DLD-1 human CC cells [12]. In their experiments, IL-4 promoted HT-29 cell proliferation for a nearly 2-fold increase after four days of treatment [12]. Koller et al. found the addition of IL-4 resulted in proliferation of HT-29 and HCT 116 CC cells [68]. In contrast, Chang et al. demonstrated that IL-4 inhibited the proliferation of HT-29 and WiDr CC cells, while it promoted cell proliferation of Burkitt’s lymphoma cell lines BL30 and BL41 [69]. Furthermore, Toi et al. found that IL-4 inhibited cell proliferation of HT-29 cells [70]. According to Topp et al. recombinant human IL- 4 had an antiproliferative effect on HTB 38 CC cells [71]. Additional studies also found discrepancies in the effect of IL-4 regarding cell growth [65,72,73]. Interestingly, when evaluating cell proliferation Lahm et al. found a significant inhibition of thymidine uptake in CC cell line LS411N by IL-4, but not by using MTT assay [65]. The different effects of IL-4 on cell proliferation in CC cell lines are summarized with regard to cell line, growth conditions and duration, ligand source and concentration, and type of proliferation assay in Table 1.

IL-4 was identified to promote EMT in CRC cell lines HCT 116 and RKO via STAT6 [74]. Koller et al. found that IL-4Rα–the subunit of type II IL-4R in epithelial cells (Figure 1)–expression promoted tumor growth in human CC cell lines HCT116, HT-29, DLD-1, SW480, SW620, Caco2, and HCA7, while IL-4 could only decrease apoptosis in HCT116 cells [68]. IL-2, IL-12, and IFN-alpha enhanced antibody-dependent cellular cytotoxicity (ADCC) against HT-29 cells and IL-4 could significantly suppress this effect [75]. Wersäll et al. demonstrated that pretreatment with IL-4 enhanced the ADCC activity of peripheral blood mononuclear cells (PBMCs), monocytes, and natural killer cells against SW948 CRC cells [76]. Recombinant human IL-4 inhibited IL-2-dependent activation and proliferation of human NK cells [77]. Nieroda et al. found a significant enhancement in ADCC activity against human CC lines LS174T and CBS after peripheral monocytes were pretreated with human macrophage colony-stimulating factor [78]. IL-4 could further enhance the ADCC activity on LS174T CC cells when tested with the peripheral monocytes from two donors [78]. Flieger et al. found IL-4 could reduce the IL-2, IL-12, and IFN-alpha-induced ADCC by flowcytometric cytotoxicity assay [79]. They used PKH-2 labeled HT-29 cells as target cells and PKH-26 labeled human PBMCs as effector cells [79]. IL-4 sensitized SW620 cells to radiation through inhibition of NF-κB [80]. Flieger et al. demonstrated IL-4 can decrease both epithelial cellular adhesion molecule and LewisY expression in HT-29 and LoVo cells, but not in SW480 cells [81]. In LS174T CC cells, IL-4 induced down-regulation of stem cell factor and its receptor c-kit, and inhibited proliferation induced by the factor [82]. The addition of IL-4 increased IL-8 release in CRC cell line HT115, but not in CRC cell lines HRT18 and H29/6 [83].

IL-4 could inhibit cell-cell adhesion without affecting cell proliferation in human CC cell line Colo205 [84]. IL-4 and IL-13 inhibited CC cell-cell adhesion via downregulation of E-cadherin and carcinoembryonic antigen molecules [84]. IL-4 was an inhibitor of hepatocyte growth factor, which could regulate hepatocyte growth factor-induced cell proliferation and other events like cell migration and invasive ability in CC [85]. JAK1 and JAK2 were phosphorylated and activated after IL-4 addition in human CC cell lines HT-29 and WiDr [86]. IL-4 changed the expression of integrin and decreased the lung-colonizing ability of HT-29 CC cells [87].

*IL-4 and cancer stem cells in CC:* Todaro et al. found that CC growth was determined by stem-like cells, which were characterized by the expression of CD133 and were resistant to chemotherapy due to the autocrine of IL-4 [14]. Anti-IL-4 antibodies inhibited the tumor growth in human CC cell line Caco [88]. Moreover, neutralizing of IL-4 increased the efficacy of chemotherapy and inhibited the CD133+ cell population by increasing their tendency to undergo apoptosis. [88].

Li et al. identified different IL-4/Stat6 activities in CRC cell lines using electrophoretic mobility shift assay [89]. They found HT-29 was an active Stat6 high phenotype and Caco-2 a defective Stat6 null phenotype [89]. HT-29 cells were resistant to apoptosis and showed more aggressive metastasis compared with Caco-2 cells [89]. The mechanism involved genes associated with apoptosis or metastasis, such as survivin and p53 [89].

*IL-4/IL-4R in CRC mouse models:* Addition of IL-4 improved muscle function and lifespan of CC-bearing mice [90]. Over-expression of IL-12 could inhibit IL-4 and STAT6 in human CC stem cells and inhibit the survival of CC stem cells in vitro and their tumor formation ability in mice [91]. IL-4 combined with CpG oligonucleotide activated tumor-specific Th1-type immune responses and suppressed the tumor growth in a subcutaneous tumor model of C57BL/6 (B6) mice [92]. An IL-4Rα aptamer-liposome-CpG oligodeoxynucleotide delivery system was demonstrated to enhance anti-tumor activity in CT26 tumor-bearing mice [93]. This system could deliver CpG into tumors and overcome TME immunosuppression [93]. Expression of IL-4 in the mouse CC cell line colon 26 inhibited tumor growth by inducing local tumor killing and systemic immunity in mice [94]. IL-4 gene transduced MC38 murine CRC cell line promoted a Th1-type response and tumor-specific immune responses in B6 mice [95].

*IL-4/IL-4R in CRC patients:* Six independent studies evaluated IL-4 serum levels in CRC patients. Five studies showed significantly higher levels of IL-4 in patients compared to healthy controls [96,97,98,99,100]. Additionally, Berghella et al. found IL-4 levels in the serum to be predictive for cancer staging and invasiveness [98]. Serum levels of IL-4 ≥ 431 pg/mL and IL-7 ≥ 54 pg/mL were associated with a 95% possibility of stage IV cancer [98]. Moreover, Zaloudik et al. provided evidence for a negative impact of increased IL-4 serum levels on CD8+ cytotoxic T-cells [99]. However, Kim et al. could not detect any IL-4 expression in serum, normal mucosa, or tumor tissue in 20 CRC patients with fluorescent bead-based detection assay [101].

In an immunohistochemical study including 359 CRC samples, patients who had high expression of IL-4 and IL-4R showed less lymph node metastases [47]. Mechanistically, IL-4 was shown to increase the expression of survivin (an apoptosis inhibitor) by activating STAT6 in primary CC cells derived from surgical specimens [102]. Tumor-cell-derived IL-4 also mediated apoptosis resistance in primary human CC cells and the human CC cell line T84 [103]. IL-4 autocrine increased growth and survival of primary human CC cells [103].

Following the cancer-stem-cell model, Kim et al. isolated CD133(+) and CD133(-) cancer cells from four CRC patients by MagSweeper and did whole-transcriptome analysis [104]. In their study, the expression of IL-4 gene was significantly higher in CD133(+) cells than in CD133(-) CRC cells [104]. Furthermore, cancer-initiating cells isolated from CRC patients showed weak immunogenicity in vitro because of their membrane-bound IL-4 [105]. However, IL-4 originated from tumor infiltrating lymphocytes was associated with better prognosis [106]. This was immunohistochemically assessed in 49 primary CC and 20 metastases [106].

Correale et al. studied 41 metastatic CRC patients and found that patients with higher serum IL-4 levels had a longer overall survival when they receive the anticancer vaccine of thymidylate synthase poly-epitope-peptide [107]. Evans et al. recruited 80 CRC patients prior to treatment and 38 matched controls [108]. No significant difference was found between IL-4 production in patients and controls, which were measured from the supernatants of activated PBMCs [108]. In CRC patients, long-term n-3 and n-6 essential fatty acids ingestion reduced total serum IL-4 by 69% (*p* = 0.025) after six months [109]. Three months after stop taking essential fatty acids, cytokine levels returned to pre-supplementation values [109].

Besides Table 1, which describes effects of IL-4 on cell proliferation, Supplemental Tables S2 and S3 summarize all the findings about the effects of IL-4 and IL-13 in CRC.

## 8. SNPs in IL-4/13 and Their Receptors in Gastric and Colon Cancer

By definition, a short nucleotide polymorphism has a minor allele frequency of more than 1% in at least one population [110]. SNPs play an important role in mendelian diseases and studies focused on their role on more complex disease like cancer in recent years [111]. In a matched case-control study, patients with aerodigestive tract cancers were investigated for frequency of the G2463A polymorphism of the myeloperoxidase gene [112]. The 2463G/A transition strongly reduced mRNA expression of myeloperoxidase and reduced cancer risk [112]. The mechanism is that the polymorphic site is located in an Alu element and leads to the loss of a transcription factor binding site [113]. A SNP in the KRAS 3′ untranslated region that binds to let-7 microRNA increased the risk of non-small cell lung cancer [114]. Genetic association studies about SNPs in cancers can be divided into two categories, susceptibility study and outcome study. [115].

There are a lot of articles about SNPs of IL-4/IL-13 or their receptors and association with gastric (summary in Supplemental Table S4 [45,116,117,118,119,120,121,122,123,124,125,126,127,128,129,130,131,132,133,134,135,136,137,138,139,140,141,142]) or colorectal (summary in Supplemental Table S5 [62,137,143,144,145,146,147,148,149,150,151,152,153,154,155,156,157,158,159,160,161,162]) cancer. Some of them revealed certain kinds of SNPs may serve as a risk factor or prognostic marker for each disease [150]. For example, in Swedish CRC patients, Shamoun et al. found IL-13 SNP rs1800925 is a risk factor while IL-4 SNP rs2243250 may serve as a prognostic marker especially in stage III CRC [150]. Some authors did not find any significant associations between SNPs of IL-4/IL-13 or their receptors and GC or CRC [163,164]. In addition, the results of available meta-analysis and previous systemic analysis were separately summarized in Table 2 [163,164,165,166,167,168,169,170,171,172,173,174].

Many reports about SNPs in gastric and CC that have been published were summarized in Table 2, Tables S4 and S5. However so far, no SNP of IL-4/IL-13 or their receptors managed to enter clinical routine diagnostics or even influence treatment of gastric or colorectal cancer. Perhaps further studies are needed in order to be relevant for clinical applications.

## 9. Discussion and Outlook

Multiple studies have focused on the effects of IL-4 and -13 in several epithelial cancers [175,176]. In CC, most of the results showed anti-proliferative effects. However, as summarized in Table 1, the effect of IL-4 on proliferation in CC cells is varying and may depend on multiple factors, among them cell line intrinsic differences. These results also point out that IL-4/IL-13 and their receptors can activate different signaling pathways and thus may have different biological functions in different types of human cancer cells. This knowledge is important when using neutralizing or blocking antibodies. Ito et al. found that addition of an IL-4 neutralizing antibody enhanced anti-tumor immunity and inhibited tumor growth in a mouse subcutaneous tumor model of murine CC cell line CT26 [177]. However, one has to keep in mind that systemic modulation of IL-4 as well as IL-13 signaling can also cause severe side-effects like a propensity towards helminth infections or acute gastric mucosal injury [178,179].

Chimeric proteins composed of IL-13 or IL-4 with PE were invented to target human cancer cells expressing the corresponding receptors [39,180]. These chimeric proteins were found effective on different kinds of cancer cell lines, such as pancreatic cancer, CC, and GC cell lines [180,181,182]. Human glioma cells were extremely sensitive, too [183].

A Phase 2 study was conducted on patients who had recurrent glioblastoma multiforme (GBM) [184]. The patients received circularly permuted IL-4PE first, and then surgical resection [184]. A Phase 3 clinical trial was carried out to compare intraparenchymal IL-13PE administration with an FDA-approved drug called Gliadel wafers for recurrent GBM [185]. It was found that the time-to-progression was significantly longer with IL-13PE compared to Gliadel wafers [185].

A hybrid peptide named IL-4Rα-lytic peptide could bind to IL-4Rα on pancreatic cancer cells and the lytic peptide could kill the cancer cells in vitro and in vivo [186]. IL-4R-targeted liposomal doxorubicin could be used to deliver drug to the human GBM 8401 cells in a mouse model [187]. Recombinant adenovirus, which expressed IL-13 on its surface, transferred genes to IL-13Rα2-expressing malignant glioma cells more effectively [188]. An IL-13Rα2 antibody could bind to glioma cells, inhibit tumor growth, and improve the survival time in a glioma xenograft mouse model [189]. IL-13Rα2-targeted cancer vaccines also showed effects in malignant gliomas in children, such as peptide-based cancer vaccine [190,191,192]. However, no articles about a similar vaccine were found for patients with GC or CC.

Chimeric antigen receptor T cells expressing IL-13 can kill target cells and have been studied in GBMs. For treating human glioma xenografts in rats, single intracranial injections of IL-13 “designer T cell” into tumor sites significantly increased survival [193]. However, as the researchers demonstrated, systemic administration of IL-13 “designer T cell” might be complicated by reaction against normal tissues which also express IL-13Rα1 [193]. Using a mouse model in which tumors show a growth-regression-recurrence pattern, Terabe et al. demonstrated NKT cells and IL-13, which signals through the IL-4R-STAT6 pathway, played an important role in escaping tumor immunosurveillance [194]. By using an IL-13 inhibitor (sIL-13Rα2–Fc), they found that IL-13 is essential for the tumor recurrence, while IL-4 is not [194].

Extracellular matrix and stromal cells constitute the main structure of the TME [195]. Different types of immune and non-immune cells are found in TME and with various cytokines they secrete, these drive a chronic inflammatory and immunosuppressive intra-tumoral environment [196]. Among the immune cells in the TME, macrophages are very abundant and can be found at all tumor stages, generally playing a pro-tumoral role [197]. Th2 (T helper type 2) cells could block T cell-induced tumor rejection by producing Th2 cytokines including IL-4 and IL-13, which can induce the formation of immunosuppressive type 2-polarized macrophages [198]. Reducing M2-type macrophages, increasing M1-type macrophages and switching M2 macrophages into M1 phenotype in tumors could inhibit tumor growth and metastases [199,200].

Future studies could focus on the down-stream signaling of IL-4R and IL-13R in different tumors. Since treatment of GBM using IL-4 and IL-13 immunotoxins is advanced compared to other tumors, more efforts should be taken to test these treatment options also in advanced gastrointestinal cancers.

## 10. Conclusions

IL-4 and IL-13 as well as their receptors are expressed in and play important roles for the progression of several different kinds of cancers. Results with regard to biological functions may vary among different cell lines or even the same cell line and tumor types, so cytokine treatment would have to be individually designed for each patient. Clinical trials have already proven that IL-4 and IL-13 immunotoxins are effective for GBM treatment. One possibility to include these agents soon into clinical treatment may be regional intraarterial treatment of liver metastases showing high receptor expression. With further research, it is maybe also possible to include new treatment strategies targeting the IL-4/13 signaling system in more patients including primary and metastasized GC and CC.

## Figures and Tables

**Table 1:** Effect of IL-4 on human colon cancer cell proliferation.

Cell Lines	Medium	Source	Concentration	Time	Type of Assay	Proliferation	Reference
HT-29, DLD-1	McCoy’s 5A + 10% FBS	R&D systems (Minneapolis, MN, USA)	50 ng/mL	1–4 days	Cell counting	↑	[12]
HT-29, HCT 116	DMEM + 10% FCS	Sigma (St Louis, MO, USA)	0–50 ng/mL	24 h	MTT assay	↑	[68]
HT-29, WiDr	McCoy’s 5A + 10% FBS; DMEM+ 10% FBS	Schering-Plow Research Institute (Kenilworth, NJ, USA)	0–50 ng/mL for HT-29, 0–100 ng/mL for WiDr	3 days	[^3^H]thymidine incorporation assay	↓	[69]
HT-29, WiDr	EMEM with amino acids, 25 mM HEPES, and 0.5% FBS	Schering Corp. (Kenilworth, NJ, USA)	NA	3 days	[^3^H]thymidine uptake studies	↓	[86]
Colo205	RPMI-1640 + 10% FCS, 2 mM glutamine, PS and 5 × 10^–5^ M 2-ME	Pharmingen (San Diego, CA, USA)	1–100 U/mL	1–6 days	Trypan blue viability cell counting	NS	[84]
HT-29	DMEM/F-12 + 2 mM L-glutamine (serum-free)	Biermann	100 U/mL	48 h	[^3^H]thymidine incorporation assay	NS	[87]
SW620	RPMI 1640 + 10% FBS and antibiotics penicillin/streptomycin	Sigma (St. Louis, MO, USA)	1–100 ng/mL	6 h	Colorimetric method using the CellTiter 96 AQueous One Solution Assay	NS	[80]
HT-29	DMEM + 1% FBS	Immunex Corp. (Seattle, WA, USA)	1–20 nM (about 14–280 ng/mL)	1–5 days	Cell counting, MTT assay	↓	[70]
SW1222, HT-29	RPMI 1640 + 10% FCS + sodium bicarbonate 2 g/L, sodium pyruvate 2 mM, PS and L-glutamine 1 mM	Genzyme (West Malling, UK)	1–100 U/mL	48 h	Liquid scintillation counting using [^3^H]-TdR	↓	[72]
HT-29, WiDr, SW1116, Co-115, LS411N, LS513 and LS1034 cells	1:1 mixture of DMEM and Ham’s F-12 + HEPES (10 mM), L-glutamine (2 mM), PS +1%FBS	Genzyme (Cambridge, MA, USA)	multiple concentrations including 100 U/mL (1 ng/mL)	6 days	MTT assay and incorporation of tritiated thymidine.	In HT29, WiDr, LS411N, LS513, LS1034 cells, ↓; in CO-115 and SW1116, NS.	[65]
LS513	The same as the above line	Schering Plough (Dardilly, France)	0–10 nM (about 0–140 ng/mL)	6 days	Liquid scintillation counting using [^3^H]-TdR	↓	[73]
HTB 38	NA	Immunex Corp. (Seattle, WA, USA)	0.01–10 ng/mL	10 days	Human tumor cloning assay	↓	[71]

↓: increase; ↓: decrease; RPMI-1640: Roswell Park Memorial Institute 1640 medium; DMEM: Dulbecco’s Modified Eagle Medium; EMEM: Eagle’s minimum essential medium; MTT: 3-(4,5-Dimethylthiazol-2-yl)-2,5-diphenyltetrazoliumbromid; FCS: fetal calf serum; FBS: fetal bovine serum; PS: 1% penicillin–streptomycin or penicillin 100 U/mL and streptomycin 100 μg/mL; 2-Me: 2-Mercaptoethanol; HEPES: (4-(2-hydroxyethyl)-1-piperazineethanesulfonic acid); F12: Nutrient mixture F-12; NS: not significant; NA: not available, the information is not provided in the article.

**Table 2:** Meta-analysis or combined studies about polymorphisms of IL-4/IL-13 and their receptors in gastric cancer (GC) and colon and rectal cancer (CRC).

First Author (Year)	Number of Studies/Articles	SNPs	Result
Zongjing Xie (2019) [163]	18	polymorphisms in IL-4	No significant association was found between polymorphisms in IL-4 and GC in combined analyses.
Jie Zhang (2013) [164]	8 studies about GC, 3 studies about CRC	IL-4 -590C>T (rs2243250)	No significant association was found in GC and CRC.
Young Ae Cho (2017) [165]	27	IL-4: rs2243250, rs2070874; IL-13: rs1800925, rs20541; IL-4R: rs1805010, rs1801275.	The IL-4 rs2070874 T allele was associated with an increased risk of gastrointestinal cancer. The IL-4R rs1801275 heterozygote was associated with a reduced risk of gastrointestinal cancer.
Sun Z (2014) [166]	7	IL-4 -590C>T (rs2243250)	IL-4 -590C>T polymorphism was associated with a lower GC risk under dominant model and allelic model in Caucasians.
Tie Wang (2016) [167]	9	IL-4 -590C>T (rs2243250)	IL-4 -590C>T polymorphism was not associated with the susceptibility of GC.
Zhang C (2016) [168]	11	IL-4 -590C>T (rs2243250)	IL-4 rs2243250 polymorphism was not associated with GC susceptibility.
Jia Y (2017) [169]	7 studies about GC, 4 studies about CRC for rs2243250; 2 studies about GC for rs2070874; 2 studies about GC for rs79071878.	IL-4: rs2243250, rs2070874, rs79071878	rs2243250 polymorphism was found to be associated with an increased risk of GC.
Liu Y (2018) [170]	3 studies about GC for rs2227284; 2 studies about GC for rs2243248; 16 studies about GC for rs2243250;	IL-4 -33T>C (rs2227284); IL-4 -1098T>G (rs2243248); IL-4 -590C>T (rs2243250)	IL-4 rs2243250 polymorphisms was associated with elevated GC risk in Asians.
Loh M (2009) [171]	203	225 polymorphisms across 95 genes, including IL-4 -590C>T	IL-4 -590C>T displayed conflicting effects between Asian and Caucasian populations in GC.
Mitsushige Sugimoto (2010) [172]	5	IL-4 -590C>T	The risk of gastric non-cardia cancer development was significantly associated with IL-4-590 T allele carrier status.
Huanlei Wu (2014) [173]	5	IL-4 -524C>T	IL-4 -524C>T polymorphism was not associated with an increased CRC susceptibility.

GC: gastric cancer; CRC: colon and rectal cancer; SNPs: single nucleotide polymorphisms.

## Data Availability

Not applicable.

## Supplementary Materials

The following are available online at https://www.mdpi.com/1422-0067/22/2/727/s1, Table S1: Effects of IL-4/IL-13 in GC, Table S2: Effects of IL-4/IL-13 in CRC cells, Table S3: Effects of IL-4/IL-13 in CRC mouse models or patients, Table S4: Polymorphisms of IL-4/IL-13 and their receptors in GC, Table S5: Polymorphisms of IL-4/IL-13 and their receptors in CRC.

## Abbreviations

GC	Gastric cancer
CRC	Colon and rectal cancer
IL-4	Interleukin-4
IL-13	Interleukin-13
SNPs	Single nucleotide polymorphisms
NKT	Natural killer T
IL-4R	Interleukin-4 receptors
IL-13R	Interleukin-13 receptors
Γc	IL-2Rγ-common chain
CHI3L1	Chitinase-3-like protein 1
STAT	Signal transducer and activator of transcription
CC	colon cancer
PI3K	Phosphatidylinositol 3-kinase
PTEN	phosphatase and tensin homolog
JAK	Janus kinase
FAM120A	Family with sequence similarity 120A
EMT	Epithelial–mesenchymal transition
11βHSD2	11β-hydroxysteroid dehydrogenase type 2
ADCC	Antibody-dependent cellular cytotoxicity
PBMCs	Peripheral blood mononuclear cells
GBM	Glioblastoma multiforme
PE	Pseudomonas exotoxin
TME	Tumor microenvironment
Th2	T helper type 2
