# Supplementary material for: Possible Roles of Interleukin-4 and -13 and Their Receptors in Gastric and Colon Cancer
Source: Int J Mol Sci. 2021 Jan 13;22(2):727. doi: 10.3390/ijms22020727 (PMC7828336; doi:10.3390/ijms22020727)
Supplement: Supplementary file 1 [file ijms-22-00727-s001.pdf]

Supplemental Table 1. Effects of IL-4/IL-13 in GC

Research object	Methods	Main results	Reference
CRL1739 cells, human GC specimens	Immunoprecipitation and immunoblot analysis	IL-4Rα and IL-2Rγ-common chain were expressed in CRL1739 cells and GC specimens.	42
CRL1739 cells	Protein synthesis assay using [^{3}H]leucine	One chimeric protein composed of IL-13 and Pseudomonas exotoxin was highly cytotoxic.	39
HTB-135 cells	Cell Cycle Analysis Kit (Becton Dickinson), FCM	IL-4 inhibited proliferation of GC cells by blocking cell cycle progression.	43
MKN-45 and AGS cells	Boyden chamber assays	CHI3L1 secreted by M2 macrophage could promote the metastasis of GC cell lines by binding to the IL-13Rα2 chain.	40
AGS and MGC308 cells; 100 human GC tissues	Co-immunoprecipitation assay, immunofluorescence, IHC	CD44v3 bound to both CHI3L1 and IL-13Rα2 in GC cell lines. CHI3L1 was upregulated during GC development.	32
HTB13, CRL1863, CRL1739, and KATO III cells; one primary GC cell line	Standard [^{3}H]thymidine incorporation proliferation assay, FCM	IL-4 could inhibit the growth of GC cells and this effect was positively related with IL-4R expression level of the respective cell lines.	44
507 GC patients	Tissue microarray and IHC	Overexpression of IL-13Rα2 chain in GC tissues was associated with poor prognosis after gastrectomy.	15
30 biopsies from GC patients	IHC	The expression of IL-4 was higher in stages I and II GC than in stages III and IV.	46
17 patients and 30 healthy controls	sandwich ELISA kit (Affymetrix eBioscience)	Serum IL-4 levels in patients were significantly higher than in controls.	45
25 patients and 54 healthy controls	Bio-Plex human cytokine assay (Bio-Rad)	Cytokine levels of IL-4 and IL-13 in patients' plasma were significantly higher than in controls.	41

Cell lines mentioned in this table are all human GC cell lines unless otherwise indicated. GC: gastric cancer; IHC: immunohistochemistry; CHI3L1: Chitinase 3-like protein 1; ELISA: enzyme-linked immunosorbent assay.

Supplemental Table 2. Effects of IL-4/IL-13 in CRC cells

Research object	Methods	Results	Reference
LS174T and HT-29 cells	WB, RT-PCR	IL-4 and IL-13 increased mucin 2 expression in LS174T cells, but not in HT-29 cells.	48
HT-29 cells	WB, RT-PCR, Northern blot; L-[^{14}C]valine	IL-13 could inhibit the macroautophagy pathway via the activation of the class I PI3K.	49
HT-29 cells	WB; [^{14}C]valine	Overexpression of tumor suppressor phosphatase and tensin homolog could counteract the IL-13 down-regulation of macroautophagy.	50
Colo201 and Colo205 cells	Protein synthesis assay using [^{3}H]leucine	One chimeric protein composed of IL-13 and Pseudomonas exotoxin was highly cytotoxic.	39
HT-29 and WiDr cells	WB	IL-13 induced phosphorylation of JAK2, JAK1, and Tyk2. IL-13 and IL-4 could induce phosphorylation of STAT6.	51
HT-29, CaCo-2, DLD-1, T84 cells	FCM, Northern blot, IP	IL-4 and IL-13 could upregulate expression of CD44.	52
RKO and SW480 cells	MTT, transwell assay, RT-PCR, WB, luciferase assay	Propofol suppressed cell proliferation and IL-13 induced EMT in CRC cell lines. Propofol suppressed IL-13/STAT6 signaling pathway.	54
HT29, SW480, Caco2 cells	WB, PCR, transwell assays, ChIP, luciferase reporter assay	IL-13 promoted EMT and aggressiveness of CRC through IL-13Rα1/STAT6/ZEB1 pathway.	55
HT-29, WiDr, SW1116, Co-115, LS411N, LS513 and LS1034 cells	MTT assay and incorporation of [^{3}H]-TdR; FACS; northern blot; biotin-labeled IL-4 binding studies	In HT29, WiDr, LS411N, LS513, LS1034 cells, IL-4 inhibited proliferation; in CO-115 and SW1116 cells, no difference. Three cell lines (HT29, LS1034, WiDr) exhibited relatively high density of the IL-4R and 4 (Co-115, LS4llN, LS513, SW1116) had lower density.	65
HT-29 and DLD-1 cells	Cell proliferation was determined by counting the cells.	IL-4 and IL-13 increased nicotinamide adenine dinucleotide phosphate oxidase 1-related proliferation.	12
HT-29 and WiDr cells	[^{3}H]-TdR incorporation assay	IL-4 inhibited cell growth of CRC cells.	69
HT-29 cells	Cell counting or colorimetric MTT assay.	IL-4 inhibited the growth of HT-29 cells.	70
HTB 38 cells	Human tumor cloning assay	IL- 4 showed antiproliferative activity.	71
LS531 cells	[^{3}H]-TdR incorporation assay	IL-4 inhibited cell proliferation.	73
HT-29 cells	FACS. PBMCs were separated from a patient with chronic lymphocytic leukemia.	IL-4 significantly suppressed IL-12, IL-2 and IFN-alpha enhanced ADCC against HT-29 cells.	75

SW948 cells, human PBMCs, mAb 17-1A	PBMCs were incubated for 0-24 h with IL-4 before ADCC assay	Pretreatment with 1 ng/ml IL-4 for 2 hours induced a significant increase in the ADCC activity of PBMCs.	76
LS174T cells; human peripheral monocytes	24-h ^{111}In-release assay was used to measure ADCC activity.	IL-4 and macrophage colony-stimulating factor had a synergistic enhancement effect of monocyte-mediated ADCC on LS174T cells.	78
HT-29 cells, PBMCs	Flow cytometric cytotoxicity assay	IL-4 decreased ADCC and reduced the IL-2, IL-12, and IFN-alpha-induced ADCC.	79
SW620 cells	ELISA	IL-4 sensitized SW620 cells to radiation by inhibiting NF-kappaB.	80
HT-29, LoVo and SW480 cells	Indirect immunofluorescence, FACS	IL-4 could decrease epithelial cellular adhesion molecule and Lewis^{Y} expression in HT-29 and LoVo cells, but not in SW480 cells.	81
LS174T cells	Northern blot, RT-PCR, FCM	IL-4 inhibited the expression of stem cell factor and its receptor c-kit in LS174T cells. IL-4 could also inhibit stem cell factor-induced proliferation.	82
HRT18, H29/6 and HT115 cells	ELISA	The addition of IL-4 increased IL-8 release in HT115 cells, but not in HRT18 and H29/6 cells.	83
Colo205 cells	FCM, RT-PCR analysis	IL-4 inhibited cell-cell adhesion but not cell proliferation. IL-4 and IL-13 could down-regulate E-cadherin and carcinoembryonic antigen molecules.	84
Primary CRC cell lines CoSp, Cope, and CoDo; HT-29 cells	WB, RT-PCR, transwell assay	IL-4 was an inhibitor of hepatocyte growth factor and it could regulate hepatocyte growth factor-induced cell proliferation and other events of tumor progression.	85
WiDr and HT-29 cells	[^{3}H]-TdR uptake studies, IP and WB, northern analysis	The addition of IL-4 phosphorylated JAK1, JAK2, and Tyk2, and activated JAK1 and JAK2.	86
Caco cells	RT-PCR, MTT assay, trypan blue test, ELISA	Anti-IL-4 antibody inhibited the growth of the Caco cells. Neutralizing of IL-4 increased the efficacy of chemotherapy and inhibited the CD133+ cells.	88
HT-29 and Caco-2 cells	RT-PCR, FCM, transwell migration assay	IL-4/Stat6 activities correlated with apoptosis and metastasis in colon cancer cells.	89

Cell lines mentioned in this table are all human colon cancer cell lines unless otherwise indicated. CRC: colon and rectal cancer; WB: Western blot; RT-PCR: reverse transcription-polymerase chain reaction; PI3K: phosphatidylinositol 3-kinase; JAK: Janus kinase; STAT: signal transducer and activator of transcription; FCM: flow cytometry; MTT: 3-(4,5-Dimethylthiazol-2-yl)-2,5-diphenyltetrazoliumbromid; ChIP: chromatin immunoprecipitation; IP: immunoprecipitation; EMT: epithelial–mesenchymal transition; ZEB1: Zinc finger E-Box binding homeobox 1; FACS: fluorescence-activated cell sorting; IFN: interferon; ADCC: antibody-dependent cellular cytotoxicity; PBMC: peripheral blood mononuclear cell; PKH: Paul Karl Horan; ELISA: enzyme-linked immunosorbent assay; NF-κB: nuclear factor kappa-light-chain-enhancer of activated B-cells; EpCAM: epithelial cellular adhesion molecule.

Supplemental Table 3. Effects of IL-4/IL-13 in CRC mouse models or patients

Research object	Methods	Results	Reference
KM12, KM12C and KM12SM cells, nude mice and 80 CRC patient tissues	ELISA, WB, IHC, FCM	High expression of IL-13Rα2 in KM12 cells was associated with liver metastasis in nude mice. IL-13Rα2 overexpression in CRC tissues was associated with later stages and poor prognosis.	13
Tissues from 359 patients	IHC	High expression of IL-4, IL-4R, and IL-13R was associated with a lower frequency of lymph node metastases.	47
KM12SM, SW620, a metastasis model of nude mice	WB, IHC, IP, transwell assay	IL-13Rα2 induced activation of the FAK and PI3K/AKT/mTOR pathways and this was mediated by FAM120A.	11
SW480 cells and murine CC cells CT26, murine liver metastasis model	WB, quantitative RT-PCR, MTT assay, colony formation assay; nude mice	IL-13 induced the expression of 11βHSD2 in CRC cells through IL-13Rα2 and promoted the malignancy of CRC.	56
KM12SM, SW620, RKO cells, mouse models	Mass spectrometry analysis, WB, IP, MTT assays, FCM	Protein tyrosine phosphatase-1B could mediate IL-13-induced cancer cell proliferation, migration, invasion and survival.	57
LS174T, LOVO, SW480 and COLO 205 cells; 25 CRC patients	ELISA, real-time PCR, WB, IHC	IL-4 and IL-13 both up-regulated the expression of chemokine eotaxin-2 in LS174T and LOVO cells.	58
Mouse model of CRC induced by injection of azoxymethane; HT29 cells	WB, IHC, MTT assay, Bio-Plex Pro Mouse Cytokine 23-Plex Panel	The formation of CRC was more frequent in obese mice than wild type mice. Silencing IL-13Rα1 inhibited IL-13-induced proliferation in HT29.	59
Murine CRC lung metastasis model; murine CC cell line CT26	FCM, BALB/c mice and CD1-knockout mice	The development of lung metastases could be significantly decreased by an IL-13 inhibitor, but not by inhibiting IL-4.	60
KM12SM and SW620 cells, nude mice and Balb/c mice	Competition ELISA, MTT assay, wound healing assay, transwell invasion assay, FCM	IL-13Rα2 D1 peptide inhibited migration, invasion, and proliferation in metastatic CRC cells treated with IL-13. Nude mice treated with the enantiomer D-D1 peptide showed a significantly survival increase to metastasis.	61
IL-4Rα and IL-13 transgenic mouse models, CRC patients and controls	IHC, case–control epidemiology study, FCM, multiplex immunoassay	Reduced IL-4R signalling increased CRC risk but reduced tumor progression. It had no effect on CRC mortality.	62
241 patients with CRC	ELISA	Patients with advanced stage of CRC had lower serum IL-13 levels. Low serum IL-13 was associated with poorer prognosis.	63

Fecal protein from 20 CRC patients and 20 healthy controls	ELISA, bicinchoninic acid assay	The expression of IL-13 was significantly higher in CRC patients.	64
Murine CC cell line CT26 tumor-bearing mice	ELISA, quantitative RT-PCR	Protein levels of IL-4, and IL-13 were higher in the serum or the tumor homogenates of CT26 tumor-bearing mice.	66
Patients with metaplastic polyps, adenomas, or carcinomas	IL-4R was detected by IHC	The expression of IL-4R was positive in all polyps (5/5), adenomas (15/15) and in 40/45 carcinomas.	67
HCT116, HT-29, DLD-1, SW480, SW620, Caco2 and HCA7 cells; IL-4Rα -/- mice	MTT assay, RT-PCR, WB, FCM, immunostaining	IL-4Rα expression promoted tumor growth in HCT116, HT-29, DLD-1, SW480, SW620, Caco2 and HCA7 cells, but IL-4 could only decrease apoptosis in HCT116 cells.	68
HCT116 and RKO cells; subcutaneous xenograft mouse models, 218 human CRC samples	WB, qRT-PCR, luciferase assay, chromatin IP assay, transwell invasion assay, IHC	IL-4 could promote EMT of HCT116 and RKO cells through E2F1/SP3/STAT6 axis. Analysis of clinical CRC samples showed a positive correlation between E2F1, SP3 and STAT6.	74
HT-29 cells and a metastasis model in nude mice	FCM, northern blot	IL-4 changed the expression of integrin and decreased the lung-colonizing ability of HT-29 cells.	87
Primary human CC cells; nude mice	FCM, IHC, immunofluorescence analysis, WB	CC growth was dictated by stem-like cells which were resistant to chemotherapy due to autocrine of IL-4.	14
CC-bearing mice	qRT–PCR, ELISA, immunofluorescence	Addition of IL-4 improved muscle function and lifespan of CC-bearing mice.	90
HCT116 cells; NOD/SCID mice; CC tissues from 40 patients	RT-PCR, IHC, WB, FCM, transwell migration assay	Over-expression of IL-12 could inhibit the expression of IL-4 and STAT6 in CSCs and inhibit their survival.	91
MC38 murine CC cell line, mice	IHC, 4-h 51Cr-release assays, female C57BL/6 (B6) mice	IL-4 combined with CpG oligonucleotide could suppress tumor growth by activating Th1-type immune responses.	92
Murine CC cell line CT26 tumor-bearing mice	IHC, FCM, qRT-PCR	An IL-4Rα aptamer-liposome-CpG oligodeoxynucleotide delivery system had enhanced anti-tumor activity.	93
Murine CC cell line colon 26; BALB/c mice	IL-4 overexpressing colon 26 cells; ELISA; 51Cr release assays	Overexpression of IL-4 in colon 26 cells could induce local tumor killing as well as systemic immunity in mice.	94
MC38 murine CC cell line, female B6 mice	Subcutaneous tumor models of mice; 4-h 51Cr-release assays	IL-4 overexpression MC38 cells promoted a tumor-specific Th1-type response in B6 mice.	95

66 patients with CRC and 87 healthy controls	multiplex bead array immunoassay (Millipore)	Serum levels of IL-4 were higher in CRC patients.	96
77 CRC patients, 70 healthy controls	ELISA	Serum IL-4 levels of the patients were significantly higher than the control group.	97
105 CRC patients	ELISA	The serum levels of IL-4 can be used as a marker of pre-invasive to invasive CRC.	98
10 CRC patients with liver metastases and 5 healthy controls	Flow cytometric immunophenotyping	Higher serum levels of IL-4 were found in patients than controls. The CD8+ cytotoxic cells might be negatively regulated by serum IL-4.	99
99 CRC patients and 107 healthy controls	ELISA	IL-4 serum levels were significantly higher in patients.	100
20 CRC patients	fluorescent bead-based detection assay, Multiplex kit	No IL-4 expression was detected in serum, normal mucosa or tumor tissue.	101
Primary CC cells isolated from 18 patients; nude mice	RT-PCR, WB, FCM, transwell assay, FCM, TUNEL staining	IL-4 could increase the expression of survivin by activating STAT6.	102
Primary human CC cells and T84 cells	FCM, IHC, Real-time PCR analysis	Tumor-cell-derived IL-4 enhanced apoptosis resistance in primary human CC cells and T84 cells.	103
Primary and metastatic tumors from four CRC patients	MagSweeper, whole-transcriptome analysis with RNA-Seq	The expression of IL-4 was significantly up-regulated in CD133(+) cells compared to CD133(-) CRC cells	104
Primary CRC cells, NOD/SCID CB-17 mice	Immunofluorescence analysis, ELISA, subcutaneous tumor model of mice	Cancer-initiating cells associated IL-4 was responsible for weak immunogenicity *in vitro*.	105
49 primary CC and 20 metastases	IHC	Expression of IL-4 by > or = 20% of CC tumor infiltrating lymphocytes was associated with better prognosis.	106
41 metastatic CRC patients	Bio-Plex human cytokine multiplex kits (Bio-Rad Inc.), FACS, IHC	The patients with higher serum IL-4 levels have longer overall survival when receiving the thymidylate synthase poly-epitope-peptide anticancer vaccine.	107
80 CRC patients and 38 matched controls	Human Th1/Th2 Cytokine Kit II (BD Biosciences)	IL-4 was measured from the supernatants of activated peripheral blood mononuclear cells. No significant difference was found.	108
Patients with CRC	ELISA, EFA ingestion	Long-term EFA ingestion could reduce total serum IL-4 by 69% after 6 months.	109

Cell lines mentioned in this table are all human CC cell lines unless otherwise indicated. CRC: colon and rectal cancer; CC: colon cancer; FCM: flow cytometry; ELISA: enzyme-linked immunosorbent assay; WB: Western blot; IHC: immunohistochemistry; FAM120A: family with sequence similarity 120A; RT-PCR:

reverse transcription-polymerase chain reaction; MTT: 3-(4,5-Dimethylthiazol-2-yl)- 2,5-diphenyltetrazoliumbromid; 11βHSD2: 11β-hydroxysteroid dehydrogenase type II; IP: immunoprecipitation; qRT-PCR: quantitative real-time PCR; NOD/SCID: nonobese diabetic/severe combined immunodeficiency; CSC: cancer stem cell; FACS: fluorescence-activated cell sorting; Th: T helper; EFA: essential fatty acids.

Supplemental Table 4. Polymorphisms of IL-4/IL-13 and their receptors in GC

First author (year)	Country	Ethnicity	Number of cases/controls	SNPs	Result
El-Omar (2003) (116)	USA	Mixed	112/209	IL-4 (-590 C/T), rs2243250	No significant association
Wu (2003) (117)	China	Asian	220/230	IL-4, rs2243250; IL-4R, rs1805010; IL-4R, rs1801275	Higher risk of developing diffuse type or cardia type cancer was observed for the CT/CC genotype of IL-4 at the position -590 (rs2243250).
Lai (2005) (118)	China	Asian	123/162	IL-4 (-590), rs2243250	No significant association
Garcia-Gonzalez (2007) (119)	Spain	Caucasian	404/404	IL-4 (-590 C>T), rs2243250	No significant association
Crusius (2008) (120)	10 European countries	Caucasian	235-244/1107-1160	IL-4, rs2243250; IL-4, rs2070874; IL-4R, rs1805010; IL-4R, rs2057768	Rs2057768 is related with non-cardia gastric adenocarcinoma.
Zambon (2008) (121)	Italy	Caucasian	144/171	IL-4 -588C>T, rs2243250; IL-4RA Ex5+14A>G, rs1805010; IL-4RA Ex11+828A>G, rs 1801275	No significant association
Ando (2009) (122)	Japan	Asian	330/190	IL-4 -590C/T; IL-4Rα Ile50Val	No significant association
Ko (2009) (123)	Korea	Asian	78-81/320-324	rs2070874; rs2243250	No significant association
Wu (2009) (124)	China	Han Chinese population	1042/1099	rs2070874	Compared with the IL-4 -168TT genotype, heterozygous -168TC and combined -168TC/CC genotypes were associated with a significantly decreased GC risk.
Pan (2014) (125)	China	Asian	308/307	rs2243250	No significant association

Yin (2015) (126)	China	Asian	234/465	IL-13, rs1800925	No significant association with gastric cardiac adenocarcinoma risk
Cárdenas (2018) (45)	Colombia	Mixed	15-17/20-30	IL-4 -590 (C/T), IL-4 Ile50Val, IL-4 Q576R	No significant association
Martínez-Campos C (2019) (127)	Mexico	Mixed	124/125	IL-4-590C/T (rs2243250)	No significant association
He B (2019) (128)	China	Asian	479/483	IL-4 rs2243248; IL-4 rs2070874; IL-4R rs2057768; IL-4R rs2107356; IL-4R rs1805015; IL-4R rs1801275	No significant association
Yun Y (2017) (129)	China	Asian	340/364	IL-4 rs2243250 (590 C/T), rs2227284 (107 T/C), rs2070874 (168 T/C) and rs1801275 (576 Q/R)	The IL-4 rs2243250 CC genotype and CT+CC genotype were associated with higher GC risk.
Burada F (2012) (130)	Romania	Caucasian	105/242	IL-4R −3223C→T, rs2057768	IL-4R −3223C→T polymorphism may increase the risk of gastric adenocarcinoma, mainly for the noncardia type.
Xia HZ (2012) (131)	China	Asian	308/425	IL-4R, rs2107356(G>A)	A significant reduction of the IL-4R AA genotype in GC risk was found when compared to GG genotype.
Bhayal AC (2015) (132)	India	Asian	182/326	IL-4 intron 3 variable number of tandem repeat (VNTR)	The study revealed an association of 2R allele and 2R carrier genotypes in the etiopathogenesis of GC.
Sampaio AM (2015) (133)	Portugal	Portuguese	100/50	IL-4 (-1098T>G), rs2243248; IL-4 (-590C>T), rs2243250; IL-4 (-33C>T), rs2070874	IL-4-590TT and IL-4-1098GG were found associated with intestinal type GC and diffuse type GC, respectively. IL-4 TTT haplotype was linked with both intestinal and diffuse type GC groups.
Seno H (2007) (134)	Japan	Asian	100/93	IL-4 (11 SNPs), IL-4R (22 SNPs)	The IL-4 gene diplotypes are negatively associated with the risk of developing GC due to Helicobacter pylori infection.

Wang Y (2017) (135)	China	Asian	362/384	IL-4, rs2243248 (-1098 G/T), rs2227284 (-33 C/T), rs2243250 (-589 T/C) and rs2070874 (-107 T/C)	The TC and CC genotypes of rs2243250 were associated with an increased risk of GC. The TTTT haplotype revealed a reduced risk of GC.
Pavithra D (2018) (136)	India	Asian	200/400	IL-4C-590-T	No significant association
Cavalcante (2017) (137)	Brazil	Mixed	119/474	IL-4, rs79071878, VNTR	rs79071878 was positively associated with the development of GC.
Sarah Yang (2017) (138)	Korea	Asian	368-377/736-754	IL-4R (rs7205663 and rs1805010); IL-13 (rs6596090, rs20541)	No significant association
Wang YM (2016) (139)	China	Asian	132/1198	IL-4R rs2057768	No significant association
Schmidt HM (2011) (140)	Singapore	Asian	60/162	IL-13, rs1800925; IL-4, rs2070874; IL-4R, rs1801275.	No significant association
García-González MA (2012) (141)	Spain	Caucasian	380/-	IL-4, rs2243250	Not relevant in determining the prognosis of gastric adenocarcinoma
Talebkhan Y (2017) (142)	Iran	Caucasian	31/46	IL-4 C-590T	Serum antibodies against Helicobacter pylori neutrophil activating protein in carriers of IL-4 C-590T genetic polymorphism amplify the risk of GC.

GC: gastric cancer; SNPs: single nucleotide polymorphisms.

Supplemental Table 5. Polymorphisms of IL-4/IL-13 and their receptors in colorectal cancer (CRC)

First author (year)	Country	Ethnicity	Number of cases/controls	SNPs	Result
Walczak A (2011) (143)	Poland	Caucasian	150/170	IL-13, rs1800925	The CT genotype is connected with a higher risk of colon cancer occurrence.
Ibrahimi M (2019) (144)	Iran	Iranian	123/152	IL-4 VNTR, RP1/RP1, RP1/RP2	No significant association
Landi S (2007) (145)	Spain	Caucasian	377/326	IL-4 (-588C>T, Ex1-168G>A); IL-4R (I75V, C431R, S436L, S503P, Q576R)	The homozygotes for IL-4 -588C>T or for Ex1-168G>A showed an increased risk for colon cancer. Women showed an increased risk associated to the IL-4 rare alleles.
Lee YS (2009) (146)	Korea	Asian	170/130	IL-4R 1902	The IL-4R 1902*T allele was found to be associated with an increased risk of colon cancer and rectal cancer, while IL-4R 1902*C allele was associated with a decreased risk.
Cavalcante GC (2017) (137)	Brazil	Mixed	63/474	IL-4, rs79071878, VNTR	No significant association
Jose M Cozar (2007) (147)	Spain	Caucasian	96/176	IL-4-590 C>T (rs2243250)	No significant association
Suchy (2008) (148)	Poland	Caucasian	607/350	IL-4 (-590 C/T)	No significant association
Wilkening (2008) (149)	Sweden	Caucasian	308/585	IL-4-590C/T (rs2243250); IL-4R-3223G/A (rs2057768)	The rare T allele of IL-4-590 was related to a longer survival. The prognostic value of the genotypes was of borderline significance.

Levar Shamoun (2018) (150)	Sweden	Caucasian	466/445	IL-4, rs2243250; IL-4Rα, rs1801275; IL-13, rs1800925	IL-13 SNP rs1800925 is a risk factor for CRC and that IL-4 SNP rs2243250 could be a useful prognostic marker.
Yanming Yu (2017) (151)	China	Asian	513/572	IL-13: rs847 A>G; IL-13: rs848 T>G; IL-13: rs1295685 C>T	A significant antagonistic interaction was found between rs848 (G-T) and allium vegetable intake; moreover, significant combined and synergistic interactions were observed for all three SNPs and overnight meal intake.
Y Yu (2014) (152)	China	Asian	299/296	IL-4 rs2070874	No significant association
Diego Marques (2017) (153)	Brazil	Mixed	140/140	IL-4 rs79071878	Polymorphism variations in IL-4 gene was associated with increased CRC risk.
Walczak A (2012) (154)	Poland	Caucasian	191/205	IL-13 - 1112 C/T, rs1800925	The CT and TT genotypes of the IL-13 - 1112 C/T polymorphism may be connected with a higher risk of CRC.
Raul Zamora-Ros (2015) (155)	Spain	Caucasian	274/266	IL-4, rs2243250	The novel dietary inflammatory index (DII) score was inversely correlated with SNP rs2243250, and an interaction was observed with CRC risk.
Xiulin Wen (2020) (156)	China	Asian	248/463	IL-4, rs2243250 and rs2227284	rs2243250 and rs2227284 in IL-4 are associated significantly with reduced CRC risk.
Nicola Ingram (2013) (62)	United Kingdom	Caucasian	1502/584	IL-4Rα: rs1801275, rs1805015, rs1805016, rs1805013, rs1805011, rs1805010.	SNP rs1801275 was associated with increased CRC risk. Reduced IL-4R signalling was associated with increased CRC initiation and risk but reduced tumour progression and no effect on CRC mortality.
Florin Burada (2013) (157)	Romania	Caucasian	144/233	IL-4R -3223C > T	No significant association
Yannopoulos (2007) (158)	Greece	Caucasian	93/108	IL-4 (–590 C/T)	The (–590 C/T) polymorphism in the IL-4 gene is associated with increased risk for early stages of colorectal adenocarcinoma.

Bente A Talseth (2007) (159)	Poland	Caucasian	118/100	IL-4 C-589T (rs2243250)	No significant association between SNP rs2243250 and hereditary non-polyposis CRC.
Juan Sainz (2012) (160)	Germany	Caucasian	1798/1810	IL-13, rs20541	Patients harboring the IL-13_rs20541_T allele had a reduced risk of CRC.
Lin Xiao (2016) (161)	China	Asian	58/-	IL-13, 1112 C/T (rs1800925)	The studied SNP does not predict responsiveness to neoadjuvant chemoradiotherapy or prognosis of locally advanced rectal cancer.
A Ho-Pun-Cheung (2011) (162)	France	Caucasian	71/-	IL-4, rs2243250; IL-13, rs20541, rs1800925	The SNP IL-13 rs1800925 was significantly associated with rectal adenocarcinoma response to chemoradiation.

CRC: colon and rectal cancer; SNPs: single nucleotide polymorphisms.
